# Evading the annotation bottleneck: using sequence similarity to search non-sequence gene data

**DOI:** 10.1186/1471-2105-9-442

**Published:** 2008-10-17

**Authors:** Michael J Gilchrist, Mikkel B Christensen, Richard Harland, Nicolas Pollet, James C Smith, Naoto Ueno, Nancy Papalopulu

**Affiliations:** 1 The Wellcome Trust/Cancer Research UK Gurdon Institute, Cambridge University, Cambridge, CB2 1QN, UK; 2 University of California, Berkeley, Department of Molecular & Cell Biology, Life Sciences Addition #3200, Berkeley, CA 94720-3200, USA; 3 Epigenomics Project, Genopole, CNRS, Univ Evry, Tour Evry2 10eme 523 Terrasses de l'Agora, F-91034 Evry, France; CNRS UMR 8080, F-91405 Orsay, France; 4 Department of Developmental Biology, National Institute for Basic Biology, Okazaki, Aichi 444-8585, Japan; 5 Faculty of Life Sciences, Michael Smith Building, University of Manchester, Oxford Road, Manchester, M13 9PT, UK

## Abstract

**Background:**

Non-sequence gene data (images, literature, etc.) can be found in many different public databases. Access to these data is mostly by text based methods using gene names; however, gene annotation is neither complete, nor fully systematic between organisms, and is also not generally stable over time. This provides some challenges for text based access, especially for cross-species searches. We propose a method for non-sequence data retrieval based on sequence similarity, which removes dependence on annotation and text searches. This work was motivated by the need to provide better access to large numbers of *in situ *images, and the observation that such image data were usually associated with a specific gene sequence. Sequence similarity searches are found in existing gene oriented databases, but mostly give indirect access to non-sequence data via navigational links.

**Results:**

Three applications were built to explore the proposed method: accessing image data, literature and gene names. Searches are initiated with the sequence of the user's gene of interest, which is searched against a database of sequences associated with the target data. The matching (non-sequence) target data are returned directly to the user's browser, organised by sequence similarity. The method worked well for the intended application in image data management. Comparison with text based searches of the image data set showed the accuracy of the method. Applied to literature searches it facilitated retrieval of mostly high relevance references. Applied to gene name data it provided a useful analysis of name variation of related genes within and between species.

**Conclusion:**

This method makes a powerful and useful addition to existing methods for searching gene data based on text retrieval or curated gene lists. In particular the method facilitates cross-species comparisons, and enables the handling of novel or otherwise un-annotated genes. Applications using the method are quick and easy to build, and the data require little maintenance. This approach largely circumvents the need for annotation, which can be a major obstacle to the development of genomic scale data resources.

## Background

Increasing amounts of gene sequence data are being held in databases around the world and methods continue to be developed that allow us to access these data in a convenient and informative manner. At the same time, large amounts of *non-sequence *gene data are also being collected, and efforts are being made to develop methods to store, access and retrieve these secondary data. Examples of this type of data would be *in situ *expression patterns, mutant phenotypes, scientific literature and 'gene pages' in model organism databases.

We were interested in finding a way to improve access to the large numbers (20,000+) of *in situ *mRNA localisation and other images that members of the *Xenopus *community had generated. The goal was to be able to retrieve images according to gene of interest in a straightforward and useful manner. A survey of image data retrieval methods in existing public databases (see Table 1) showed that the mechanisms for retrieving image data by gene were almost invariably based on gene names or symbols, or parts of gene names. We felt that these name based databases probably required a significant annotation or curation effort to set up, and that, in general, name based methods suffer from the following drawbacks. First, such methods rely on the underlying gene annotation (process of associating a gene name with a specific sequence or genomic locus), and its quality, completeness and stability. This will be quite problematic for model organisms, like *Xenopus*, where gene annotation is not finished. And second, they probably require one to go through a process of associating an application's data-set with the correct gene name and ensuring that this is kept up to date. The problem is that gene annotation is a work in progress, both conceptually and for specific organisms, and although significant effort has been put into this over recent years (see [1] for an overview), it is clear that gene names (a) are potentially unstable, (b) can be inconsistent between organisms and (c) are not available for the many as yet unknown or novel genes, and that this is likely to remain so for some time to come. Even for known genes, extensive lists of aliases can be required to cope with the naming history of that gene and the variety of names for the orthologous genes in different species. In short, it is a relatively labour intensive approach, and will usually require ongoing maintenance. Given this, and the incomplete state of gene annotation for *Xenopus*, we decided to investigate other approaches.

**Table 1:** Analysis of access methods used by other image data providers

**database**	**function**	**access methods**	**URL**
FlyBase	ImageBrowse/Fly Express	gene name, anatomy, or development stage	

Allen Brain Atlas		gene name, accession numbers and other IDs, anatomy, or markers	

EMAP	EMAGE	gene, anatomy or development stage	

MGI		gene, anatomical structure, developmental stage, GO terms, assay type	

4DXpress		gene names, pre-computed orthologs, ontologies	

Xenbase		found on Gene pages	

UCSC	VisiGene	gene name or key word	

NIBB	WISH Photo Browser	development stage, view or clone name	

WormBase	Expression Pattern Search	cell, cell group, or life stage	

ANISEED	Expression Search Tools	development stage, or molecule ID	

ZFIN	Search for Gene Expression Data	gene name, anatomy, or development stage, and other more specific terms, indirect via BLAST	

Summary of search methods used by available public image databases for accessing images, found at the time of writing. Data gathered by visiting each database and reading associated publications.

Making the observation that *in situ *images are intrinsically associated with a sequence (the *in situ *probe), we hypothesised that a method based on sequence similarity searching might provide the power and accuracy needed, without the overhead of the annotation that would be required to create a gene name based application. If such an approach was successful, it would also generalise to other collections of data where the data are gene based and an identifiable sequence is associated with each piece of data. For example, sequence accession numbers are widely embedded in scientific literature, and to be able directly to access literature on the basis of sequence similarity would appear to be useful.

The general model of the method would be for the point of entry into the search process to be the *sequence *of the user's gene of interest. This sequence would be used to run a sequence similarity search (e.g. BLAST [2]), in the background, against a specifically prepared database of sequences associated with the target search data (such as images). The IDs of the matching sequences are linked to the target data, enabling the target data that matches the query sequence to be returned to the user. This effectively uses the query sequence as 'bait' to retrieve the non-sequence data. The signature of the method is the direct return of useful target data in response to the query sequence.

The sequence based search would retrieve data associated with sequences similar to, as well as identical to, the query sequence, and thus would enable data retrieval for related genes as well as the specific user's gene. This would have useful application in (for example) cross-species searches. Equally, there is no requirement for these to be gene sequences, and the method could be used for any type of biological data with a sequence.

A search for applications of the proposed method where it might already be in use drew a blank. Specifically we failed to find any instances of a sequence similarity search being used as the entry point for direct retrieval of image or other non-sequence gene data.

What we did find were (a) enhancement of the output of standard BLAST sequence searches with clickable links to other data where available, (b) BLAST searches leading to lists of sequences (without alignment detail) which acted as links to other data, and (c) pre-computed sequence similarity search results being used to link items within databases. The following are examples of these mechanisms. Probably the most familiar are the 'linkouts' in the results pages for NCBI BLAST [3]. These linkouts take the user to various other of the NCBI family of databases, such as the PubMed literature databases [4] and the Entrez Gene database [5]. They are undoubtedly useful, but the links must be followed separately to investigate the secondary data, and not all reported sequence matches have linkouts. One of a number of gene expression databases, 4D*Xpress *[6] has an emphasis on cross-species comparison. Primary access to the data is through gene names, plasmid IDs, and temporal and spatial expression descriptors (stage, anatomy, etc.) via ontologies. A BLAST search can be performed, taking the user to lists of either systematic gene IDs or probe sequences from which further links lead to expression data. Access to data for orthologous genes in other species is provided by pre-computed relationships (from Ensembl [7]), and also for paralogous genes within species. The zebrafish model organism database, ZFIN [8], provides a facility much like the NCBI BLAST search, where the target database is the set of gene sequences which have expression data available, and image data for each gene is accessed via linkouts. While these examples demonstrate the usefulness of using sequence similarity searches and search results to link resources, they fall short of the type of search mechanism proposed here by not proceeding directly from the query sequence to the retrieved target data.

A number of other search methods of obvious utility were implemented in the databases we looked at, including development stage and anatomy based searches, and pattern matching methods. Image retrieval was also often provided based on plasmid ID or accession number, which is, at one level, a good proxy for the gene. However, it requires a very specialised knowledge to be familiar with enough IDs for this to be really useful, and we discount this as an effective method for gene based searches.

It seems clear that building a system that is based on the gene sequence, and independent of gene annotation, should confer a number of advantages:

• no additional annotation or curation effort would be required, making applications easier to build and maintain

• the mechanism would work just as well with genes of unknown function, or genes which are un-annotated for any other reason

• data retrieval is not limited to the gene of interest but is extended to similar genes in a controlled fashion

• data retrieval would be straightforwardly cross-species, with no concerns about how gene names varied between organisms

• data retrieval would also operate across related genes with different names in the same organism

• retrieved data for multiple genes would be intrinsically self-organising on a basis familiar to molecular biologists, i.e. evolution

Data management, search and retrieval methods have been widely investigated, and are well discussed in the literature (for examples in biology see [9-16], and also [17,18] for some of the broader arguments). The topic of gene specific data retrieval has also received much attention, particularly in the context of literature searches, and the recent review by Kersey and Apweiler [19] provides a general insight into the problem. Possibly the most widely used literature search engine in biology, PubMed [4], is based on a text search; use of logical operators and a *limits *mechanism allow users to perform complex queries which can include gene and species names. Methods focused more specifically on genes, developed for literature retrieval, generally use data mining techniques to identify and disambiguate gene names and symbols in free text (see [20-23]), in order to use the text itself more effectively. iHOP [24] is another interesting example using *natural language processing *to much the same ends.

To test our hypothesis that the use of an *indirect sequence similarity search method *to retrieve gene based image data would prove effective and also generalise to other types of data, we set out to implement the method in image, literature and gene name searches.

## Results and discussion

As anticipated, building applications to use an indirect sequence similarity search was straightforward, and required minimal data preparation. The applications performed as intended, and, with a common methodology, they shared many of the same software components.

We built three applications: quickImage, an image search engine, to fulfill our original goal for this project, quickLit, to retrieve gene-specific literature, and quickGene, a tool to explore gene names. All applications work cross-species, and are available as publicly accessible resources:

quickImage 

quickLit 

quickGene 

Discussion with colleagues in the ascidian research community provided early evidence of the attractiveness of the method for accessing large collections of image data, and we were able to combine a large number of *Ciona intestinalis *images with those from *Xenopus laevis *and *Xenopus tropicalis*.

The individual applications are described below, but first we describe the method generically, to avoid repetition and to make the underlying processes clear.

### generic method for indirect sequence similarity search

#### (i) data preparation

The first step is to identify the data sources for the project, download the relevant data files, and extract the required data into a local managing database. The core part of this is the pairwise association of each piece of data with the sequence identifier(s) it is intrinsically related to, plus whatever other information is available to describe the data. Generally each source of data requires its own parser to be written, but these use simple computing tools and may be written in a few hours by a competent bioinformatician. The second step is to use the sequence identifiers to download the actual sequences from whichever database they are most conveniently available, and build a blastable database from these sequences, indexed on the sequence identifiers that have been stored in the managing database. To minimise BLAST search times care should be taken to ensure that this database only contains sequences leading to useful target data.

#### (ii) application logic

**Figure 1:**
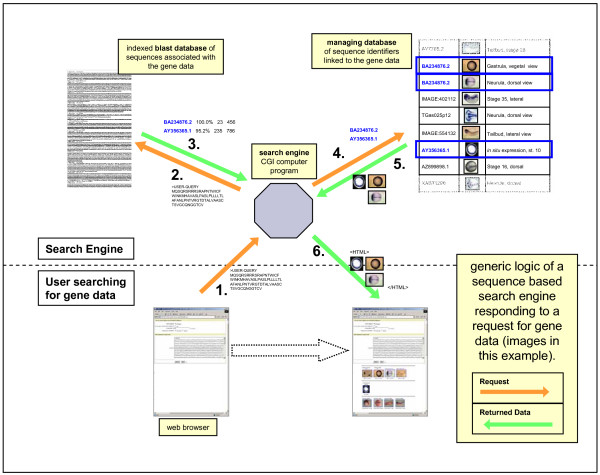
**Generic application logic used in indirect sequence similarity search for gene data**. (1.) the user pastes a gene sequence into the browser window and sends it to the search engine; (2.) the gene sequence is blasted against the database of sequences associated with the gene data; (3.) IDs of matching sequence are returned to the search engine; (4.) the matching sequence IDs are used to query the local managing database for available gene data; (5.) a list of matching gene data and descriptive text is returned to the search engine; (6.) an html formatted page containing the retrieved gene data and descriptive text is returned to the user's browser.

Once built, the application then functions in the following way. First the user submits the sequence of their gene of interest to the search engine via a web page. The search engine then uses this as the query sequence to search the blastable database. Identifiers for the matching sequences are returned to the search engine from the BLAST search, and these are used to identify the correct pieces of associated data in the managing database. These data are then returned to the user in a results web page, with whatever other information is available to make the data most useful. This is illustrated schematically in Figure 1.

The search engine applications were built from our standard programming toolkit, but could easily be written in any suitable computer programming language. The primary requirements for such an application are that it can run a system level command (BLAST), that it can interact with an SQL database on the local network, and manage internet connectivity.

Sensitivity can be adjusted, as in any BLAST search, by setting the maximum E-value for reported results, and this is provided as a control for users.

### quickImage

This application uses the query sequence to search for biological image data where the images originated from sequences the same as or similar to the query sequence.

We identified five major collections of *Xenopus *image data (see Table 2.) which included all of the large groups of *Xenopus *images known to the community. A key person for each collection agreed to facilitate the transfer and interpretation of data associated with the images, and to ensure that the images were available on a local web server. For the *Ciona *images, all the relevant data were transferred from a single source, ANISEED [25], which made the data collection and preparation steps simpler than for the *Xenopus *images.

**Table 2:** Contributing image collections for quickImage

**image collection coordinator**	**collection location URL**	**type of collection**
Richard Harland	University of California, Berkeley	*in situ *images

Nancy Papalopulu	University of Manchester, UK (unpublished images)	*in situ *images

Nicolas Pollet	Universite Paris-Sud, Orsay, France	*in situ *images

Jim Smith	Gurdon Institute, Cambridge, UK	morpholino screen with *in situ *images

Naoto Ueno	NIBB, Japan	*in situ *images

Patrick Lemaire	IBDML, Marseille	*in situ *images

The images collections and the key individual responsible for coordinating transfer of data to the image search database. A URL is given where the images are available as an existing public resource.

Each image collection provided a set of image file names and related data, and the URL of the web folder containing the images, or the images themselves. The image collections had mostly adopted internally consistent and straightforward naming convention for the image files based on plasmid or sequence IDs, and the links between images and their source sequences were straightforwardly parsed into the managing database. The Harland image collection, which had grown over a period of time with a gene based naming convention and manually edited file names, was more of a challenge, and gave us an opportunity to develop guidelines for image file naming which will help with future submissions to this project. The Harland image file names were edited to include an accession number before being added to the current version of the search engine.

The file naming guidelines can be summarised as follows. File names should be built consistently, but most consistent schemes will work; names should not include spaces or other problem characters (+, =, /, etc.); sequence information should be included in the form of the plasmid name, accession number or local ID; experimental information (stage, view, etc.) may be embedded by means of short codes or integer values; embedded information within the name should be divided by hyphens, or other characters (e.g. underscores) so long as the character used does not otherwise appear in any of the parts of the name; names should be unique within a collection. Development stages should be expressed as precisely as possible. An example of a conforming name would be BC063191-IS-12-LAT.gif (*in situ *made using probe from sequence BC063191, stage 12 embryo, lateral view).

In this application the sequences identifiers are a mix of sequence accession numbers and plasmid IDs, although this did not affect our ability to download the corresponding sequences from the appropriate databases to build the blastable database. To assist the user in identifying imperfect matches to their query sequence, we provide best BLAST hit descriptions for the originating sequences from the NCBI protein database, for human, mouse and *Xenopus*. These data are made available as part of the results.

Images already on a public web server are accessed in their original locations; otherwise they are placed in a folder on our local web server. Whichever method is used, the search engine returns HTML placeholders for the images, containing the URL of the actual image location, from which the browser then loads the image itself. This helps to reduce the load on our web server and can decrease download time for users.

In practice the search engine works well, and the user is rewarded in a few seconds with sets of images for their gene, and/or genes with similar sequences. In general there is sufficient additional information to organise the images according to the collection, the type of experiment, and the temporal progression of development stages. The user can scroll through the sets of images, and also click on any image to view it full size. The user can also search for images from any combination of the available species.

We included the ability to perform key-word searches of the BLAST identification data, and this combines in a simple Boolean AND/OR fashion with the sequence based search (i.e. results are returned from either the sequence match or the key-word match, but will not be reported twice). Thus demonstrating that the sequence based method can easily be combined with more traditional text based methods, and logical operators.

BLAST performance was very good for this application: generally < 1 second at current sequence numbers. This is primarily because the number of sequences (see below) is small for a BLAST database.

**Figure 2:**
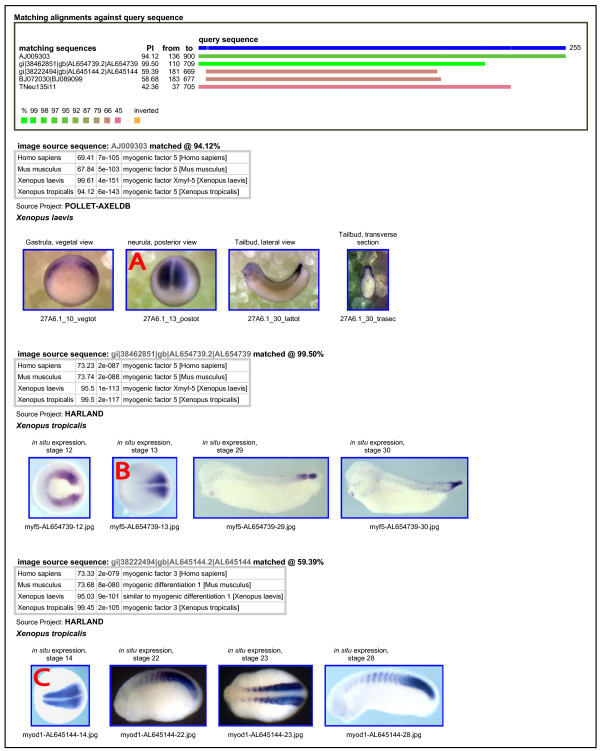
**Example output of quickImage**. The query sequence was *X. tropicalis * myf5, used to retrieve image data for this and related genes. The upper panel shows alignment and similarity between the query sequence and the matching image source sequences. The first three sets of retrieved images are shown; for each set, the accession number of the image source sequence and the best BLAST matches against human, mouse and *Xenopus *proteins are provided for identification purposes, as well as the originating image collection and species. Images marked A and B show highly similar expression of myf5 in the two frog species at the same development stage. The image marked C shows an interestingly similar expression pattern for the related gene myod/myf3 at a slightly later stage.

An example of the output of the image search engine is shown in Figure 2. The figure shows the first three sets of images retrieved by a search using *X. tropicalis *myf5 as the query sequence, and searching only *Xenopus *images. We observe reassuringly similar expression patterns for this gene at stage 13 in the two model frog species (see images A and B marked in the figure). The third set of images in the figure are for the related *X. tropicalis *gene myod/myf3, and interestingly we see a strikingly similar expression pattern at the slightly later development stage 14 (image C).

At the time of writing quickImage provides access to 39,429 images associated with 6,807 source gene sequences from three species. We invite expressions of interest from holders of other model organism image collections who feel this approach might suit their data.

### quickLit

This application uses the query sequence to search for published literature containing references to sequences the same as or similar to the query sequence. The output is lists of articles with titles, authors, etc. and links to PubMed. Application data were downloaded in various formats from NCBI GenBank [26], FlyBase [27], WormBase [28] and SGD [29], which contained links between accession numbers or other sequence identifiers and data describing published articles and their related PubMed IDs. We found a general lack of gene specific literature references for some non-vertebrate organisms in the GenBank sequence entries (which most of the data came from), hence the inclusion of the model organism databases. In general from GenBank we used the RefSeq [30] data, except for *Xenopus *(which we have a particular interest in) where we used the nr database which has better coverage. We have not attempted to ensure complete coverage of all possible publications from all possible sources.

Initial analysis of the downloaded data showed that most of the sequence identifiers were only associated with genomics papers, which would be uninformative for the intended use. These were easily identified from the data by counting sequence identifiers per PubMed ID. A paper with 100 or more sequences referenced to it was designated a genomics paper, and such papers were removed from the database, as were the sequences which were *only *referenced by genomics papers. This made the BLAST searches proportionally, and significantly, quicker. After these operations the managing database contained 116,188 sequence identifiers, and 175,128 titles or PubMed IDs.

There was clearly an imbalance in the number of citations per sequence between the model organisms (fly, worm, yeast) incorporated into the search engine from their own databases, and the other organisms sourced from GenBank alone (data not shown). The greater numbers of references from the model organisms' databases are presumably a reflection on the model organisms' specific literature curation activities, while the literature associated accession numbers from GenBank are for " [p]ublications by the authors of the sequence that discuss the data reported in the record" (GenBank help page text).

BLAST performance was somewhat slower than for the image search application, but with approximately 20× more sequences, this was to be expected. By comparison, it was still much faster than searching one of the complete protein databases (e.g. RefSeq or nr from NCBI) because of the exclusion of sequences without references or those only referenced by genomics papers.

**Figure 3:**
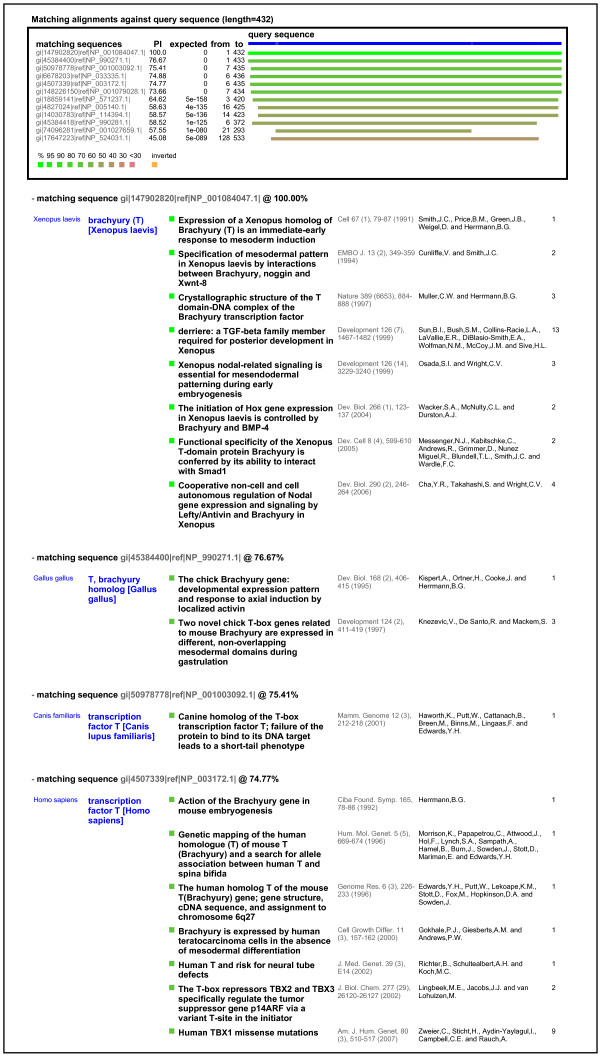
**Example output of quickLit**. The query sequence was *X. tropicalis brachyury*, used to retrieve literature references for this and related genes. The retrieved references are shown for the first few matching sequences. The retrieved data shows a high degree of apparent relevance as indicated by the title of each paper, and clear organisation of reference by species. Reference summaries and associated sequence data were downloaded from NCBI GenBank and various model organism databases.

Sample output for a typical cross-species search using the *Xenopus laevis *gene brachyury is shown in Figure 3, illustrating the high degree of relevance of the retrieved references across several common species.

The disparity in numbers of references between the model organisms' databases and GenBank suggests that there are many more accession numbers embedded in papers than currently available in downloadable databases. A project to systematically mine all biological literature for accession numbers could generate a very powerful, gene based literature search tool. This emphasises the importance of including sequence accession numbers, as well as gene names, in journal submissions.

### quickGene

NCBI's Entrez Gene [5] is a key resource for gene based data, but occasionally, when searching for named genes, we feel it would be useful to know if the lists of gene entries returned were actually related to each other. The quickGene application was designed to address this. The application uses the query sequence to search NCBI's Entrez Gene data for entries with sequences the same as or similar to the query sequence. The output is lists of genes and links to Entrez Gene. Application data were downloaded from Entrez Gene. To reduce the BLAST search times and concentrate the search on the most likely interesting results, we used only the subset of data corresponding to the better known model organisms. To further speed up the search we retained only one protein sequence per gene entry. After loading, the managing database contained ~652,000 sequence identifiers and Entrez Gene IDs.

BLAST performance was again commensurate with the numbers of sequences to be searched, and was still less than 10 seconds for a ~400 residue protein. This was a little slower than for the other two applications because of the larger numbers of sequences.

**Figure 4:**
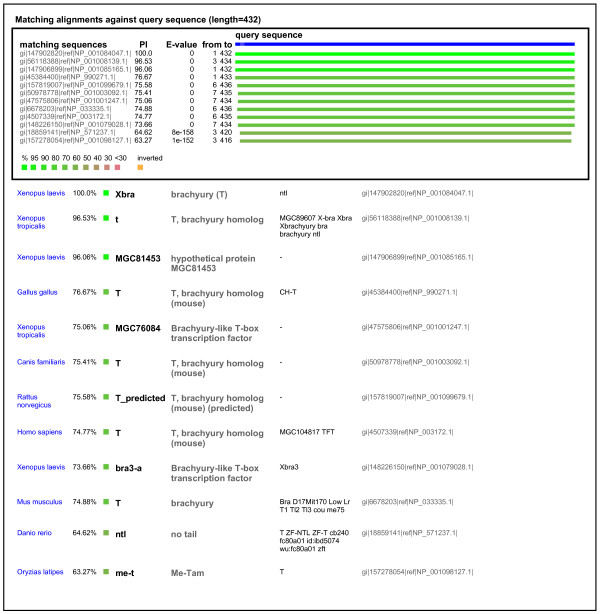
**Example output of quickGene**. The query sequence was *X. tropicalis brachyury*, used to search gene name data from Entrez Gene. Note the variable nature of the retrieved gene names for this set of related genes.

Gene names can be searched across all major model organisms, or more deeply within a single species. Sample output for a typical cross-species search using the *Xenopus laevis *gene brachyury is shown in Figure 4, and illustrates well some of the problems of gene name based systems discussed above, with four different gene names being used (plus some un-annotated 'names') in the first twelve matches.

### application performance and validation

To show that our method performs as intended we needed to demonstrate that, for a given gene of interest, an application would retrieve all the data for that gene that were available. We chose to do this with the image data, implementing a text search mechanism in quickImage to perform an internal comparison between the sequences based method and a text based method. We selected a test set of arbitrary, but developmentally interesting, *Xenopus *genes, and investigated the performance of each one individually, comparing the images returned by the two methods. This approach was necessary because, although some of the images in the database were already annotated, most of them were not, and so the 'correct' total number of image sets for each gene was not known in advance.

It was not feasible to make meaningful, large-scale, systematic comparisons with other databases providing access to similar collections of data. Other expression image databases generally had quite different collections of images from ours, although there was overlap in some cases. For the literature and gene name applications the relationship between our data sets and the databases we downloaded the data from were much clearer, but there was no practical way of generating and assessing large-scale test data in the other databases.

Images in quickImage are grouped into *image sets *according to which *image associated sequence *they were derived from, and our comparison between the two retrieval methods was based on image sets. Text data to search against was provided by the BLAST hit descriptions from the best match between the image associated sequences and NCBI protein sequences for mouse, human, *Xenopus laevis *and *Xenopus tropicalis*. Data to search with, for each test gene, was collected from the NCBI Entrez Gene database [5], and consisted of the current gene symbol and the full gene name (as well as older aliases), and, retrieved via the indicated link, the longest reference mRNA sequence.

Comparison between the two retrieval methods was made as follows. First the mRNA was used to retrieve images by the sequence method, and the number of image sets retrieved for each gene was noted. Then an exhaustive text search was performed for each gene, variously using the current gene symbol, the whole and parts of the full gene name, older aliases and other commonly used names. For many of these searches, whether sequence or text based, images for other genes were retrieved along with the target gene images, and care was taken to disambiguate search results by inspection. In no cases did the text searches turn up any additional images for the target gene that the sequence based search had missed.

The results are summarised in Table 3, and several things are clear from this comparison. First, for all genes tested, the sequenced based retrieval using the full-length mRNA found all the images in our database that could be found by trial and error combinations of text search terms. For bmp4 the reference mRNA from GenBank (via Entrez Gene) was truncated, and the full-length sequence was sourced from our own (public) *Xenopus *EST database [31]. Second, the current gene symbol failed in the majority of cases (7/12) to retrieve images for our test set with the text search, although using older aliases tended to give better results (e.g. p53 rather than tp53). Third, the text search could eventually be made to work for most genes (11/12) if sufficient alternatives were tried. This sometimes required the complete exact text of the full gene name (data not shown). Fourth, the text search (as set up here) would clearly fail for genes where the image associated sequence was not in the coding region of the gene (smarcd1), as there would be no text in the database to search against for those image sets.

**Table 3:** Comparison of text based and sequence based retrieval methods for image data for an arbitrary set of genes

				**numbers of image sets retrieved for target gene using different methods**			
					
		**test gene**		**sequence**	**text**		
	
**frog species**	**current symbol**	**current full name**	**mRNA accession or other identifier**	**with full-length mRNA**	**with current gene symbol**	**with trial and error text terms**	**notes**
*X. laevis*	**chrd**	chordin	NM_001088309	3	0	3	
		
	**hes1**	hairy and enhancer of split 1	NM_001085917	1	1	1	
		
	**nog-A**	noggin	NM_001085644	1	0	1	
		
	**Six1**	homeobox protein SIX1	NM_001088558	1	1	1	
	
*X. tropicalis*	**bambi**	BMP and activin membrane-bound inhibitor	NM_001008193	2	2	2	
	
	**bmp4**	bone morphogenetic protein 4	Xt7.1-XZT65619.5.5	3	1	3	mRNA from Entrez Gene appears to be truncated, used EST-based contig sequence instead
	
	**fgf8**	fibroblast growth factor 8	NM_001008162	1	0	1	
		
	**lhx1**	LIM homeobox 1	NM_001100228	2	0	2	
	
	**smarcd1**	SWI/SNF related, matrix associated, actin dependent regulator of chromatin, subfamily d, member 1	NM_001004862	1	0	0	probe design sequences were in 3'UTR so there were no BLAST hits for text identification
	
	**sox2**	SRY (sex determining region Y)-box 2	NM_213704	4	0	4	alias gene symbol 'sox-2' worked better than 'sox2'
	
	**t**	T, brachyury homolog	NM_001008138	6	!!	6	a large number of protein descriptions contain the letter 't'
	
	**tp53**	tumor protein p53	NM_001001903	2	0	2	older alias gene symbol 'p53' retrieved both image sets

Image sets are defined by their associated sequence and source collection. Each associated sequence has been blasted against the NCBI protein databases, retaining the best match for human, mouse and the two frog species. Text based retrieval used simple text matching (allowing wild cards) against the protein description returned by BLAST. Sequence based retrieval used BLASTn against a database of the image associated sequences. For each gene the number of images sets retrieved by the sequence method, using the full-length mRNA, was noted. Text searches with various combinations of the gene symbol, exact full name, and more commonly used names, confirmed that the sequence method appeared to have retrieved all image sets for the target gene in each case. Care was taken to disambiguate search results on percent identity or protein description (as appropriate) by inspection, where images for other genes were retrieved along with the target gene images.

The relatively poor performance of the gene symbol in the text based search is attributable to the use of the BLAST hit descriptions as the text data to search against. These are generally close to the *full name *for the gene/protein, and may only contain the gene symbol by chance. Whilst this does mean that the full name may often work with text based retrieval, the full name for many genes may be difficult to recall exactly (e.g. "SRY (sex determining region Y)-box 2" for *Xenopus tropicalis *sox2), and even small differences may make the search fail.

Our test showed that the sequence based retrieval method works very well, and, although this is a relatively small number of genes, we have no reason to believe that the results are not broadly representative. The method is clearly not infallible. Searches will fail if there is no (or minimal) sequence overlap between the user's query sequence and the data-associated sequence for a given gene. Chances of this can be minimised by use of the longest appropriate sequences, both by the user as the query sequence, and by the application builder in the BLAST database of data associated sequences.

One of the great strengths of the method is the ability to search across different species for data from orthologous genes, without being affected by inconsistent gene naming schemes. This is also true for related genes within a species (for example, the Sox, HMG-Box and other families of transcriptions factors in human share sequence similarity). Furthermore, the retrieved data can be usefully ordered according to the approximate evolutionary relationship of the genes involved. The choice of mRNA or protein sequences to search with will affect the depth of detectable homology, as expected in a BLAST search, and may affect the range of genes that data is returned for. The retrieval and organisation of data for related genes can be seen quite clearly in Figures 2, 3, and 4.

Unknown or novel genes with typically uninformative database descriptions like 'hypothetical protein LOC23277' or 'novel zinc finger protein' are unlikely to be found usefully with a text search, unless these highly specific terms are known or very broad definitions needed. By contrast, the sequence based retrieval method works equally well with novel genes as with known genes. Of the 2,618 sequences underlying the *Xenopus *image data, 128 (5%) had only uninformative identifying best hit BLAST descriptions (in human, mouse, *X. laevis *and *X. tropicalis*), and are likely novel genes.

### limitations of this approach

The main limitations of this approach are the requirement for the user to acquire a sequence for the gene of interest before they can do the search, the need to interpret the results sufficiently to distinguish between the gene of interest and related genes (and between actual orthologs and other homologs, across species), and the inability to search with standard gene symbols.

In addition, something that would need careful consideration for any implementation of our method is the impact on the overall search time of the BLAST search. BLAST search times are dependent on the size of the BLAST database, and for larger databases may eventually go beyond that which most users will cheerfully wait for. Depending on the importance of the data being sought, and the application strategies adopted to mitigate search times, this may become an issue. Application developers will need to evaluate typical BLAST search times for proposed databases, and ensure that the anticipated number of *concurrent *users does not overload the available hardware and create unacceptable delays in running searches.

## Conclusion

Motivated by the need to provide access to large numbers of images accumulated by the *Xenopus *community, we have developed a novel approach to the problem of biological image data management. In the process we discovered that applying the same methodology to other sets of data can lead to effective search applications in different fields, particularly in literature searches where retrieved references show a high degree of relevance and are intrinsically well organised. We believe the method will have application to almost any collection of sequence based data, and will usefully extend the available repertoire of search tools and methods.

The advantages of this method of indirect sequence based retrieval are its independence of gene annotation, the ease of making cross-species comparisons, the elimination of the trial and error associated with gene name based systems, the accessibility of novel or otherwise un-annotated genes, the organisation of retrieved data in an intuitively obvious way, and the ability to build applications simply and quickly, with low maintenance overheads.

We suggest some other potential applications of the method. Firstly, it may be useful as an alternative way to access gene lists in (say) model organism databases, especially for researchers unfamiliar with a species and its gene naming conventions. Second, accession numbers provide the link between the gene data and the sequence, and many of these have associated Gene Ontology (GO) terms [9] available in public databases. This presents an opportunity to combine the power of ontology based queries with the simplicity of the sequence retrieval method. Third, the relatively future-proof mechanism of sequence based searching may appeal to community based projects like the proposed Gene Wiki [32] that rely on "small contributions from a large population of contributors" and may not have the resources to establish an ongoing maintenance programme. In a more general sense there are no obvious technical obstacles to incorporating the method into existing search interfaces for use where gene data is being sought.

## Methods

### acquiring and storing data

For quickImage, image related data were acquired directly from the image collections, usually in the form of a spreadsheet. This was parsed into a uniform SQL database format using simple computing tools (grep, SQL, etc.) as appropriate. One of the projects was already managed locally (Smith morpholino screen) and the data were simply transferred from one part of our database to another. For the other two applications, data files were downloaded by ftp from the various source databases and parsed in much the same manner as the image data.

### downloading source sequences

For quickImage, lists of sequence accession numbers were uploaded to Entrez using the Entrez batch tool [33], and the returned sequences were downloaded directly into a fasta file. Other sequences were available internally from our EST database [31]. cDNA sequences were used in preference to pairs of 5'/3' EST sequences from the same clone. Where a pair of ESTs was used the two sequences were merged if there was a detectable overlap, otherwise they were simply joined end to end with a poly-n linker between them. For the other two applications, it was generally more effective to download and manage a local copy of the NCBI 'nr' protein database, and then use the NCBI utility 'fastacmd' to extract unique sequence entries with lists of 'gi' numbers and the -t option from a blastable database of the nr sequences. Some work had to be done to remove duplicate entries, particularly from the literature data sets which were downloaded from several sources.

### BLAST

A blastable database of the sequences associated with the data to be retrieved was made for each application. Multiple databases (for example in quickImage, where there was a database per image collection) were joined using a .nal file to create a single effective database.

The user BLAST searches are run using blastn, tblastn, blastp or blastx as appropriate, allowing the user to set the maximum E-value and limit the number of returned hits, and using low-complexity filtering "for look-up table only". Tabular output is requested.

### CGI program

Details of the design and function of the computer program are not presented here, as they contain nothing particularly novel or unusual; the underlying ideas are not dependent on implementation detail, and the primary functional requirements (see above) are available in most web programming environments. Interested readers may view or download the source code from the project home page (see Availability and requirements, below). A zipped archive of the code at the time of writing is available as Additional file 1.

## Availability and requirements

Project name: **quickApps**

Project home page: 

Operating system(s): Windows 2000 and XP

Programming languages: C++, SQL

Other requirements: Apache, NCBI BLAST, SQL-Server, DOS

Source code: can be viewed and downloaded at the project home page

License: code is freely available under a BSD style license

## Authors' contributions

MG devised the underlying ideas in discussion with NP_a _and JS, performed most of the computational work and drafted the manuscript. MC identified, downloaded and extracted the data for quickLit. RH, NP_o_, JS, NU and NP_a _provided access to and coordinated data transfer for the image collections under their control. RH, NP_o_, JS, and NP_a _contributed to the manuscript. RH helped develop the image file naming guidelines. All authors read and approved the final manuscript.

## Supplementary Material

Additional file 1 archive of code for the applications described in the manuscript. quick-release-archive. Click here for file
