# Supplementary material for: Evading the annotation bottleneck: using sequence similarity to search non-sequence gene data
Source: BMC Bioinformatics. 2008 Oct 17;9:442. doi: 10.1186/1471-2105-9-442 (PMC2587480; doi:10.1186/1471-2105-9-442)
Supplement: Additional file 1 — archive of code for the applications described in the manuscript. quick-release-archive. [file 1471-2105-9-442-S1.zip › quick-release-code/apache/htdocs/quickGene/index.html]

quickGene


|  |  |  |
| --- | --- | --- |
|  |  |  |
| >NP\_005441.1 noggin precursor [H.sapiens]   MERCPSLGVTLYALVVVLGLRATPAGGQHYLHIRPAPSDNLPL VDLIEHPDPIFDPKEKDLNETLLRSLLGGHYDPGFMATSPPED RPGGGGGAAGGAEDLAELDQLLRQRPSGAMPSEIKGLEFSEGL AQGKKQRLSKKLRRKLQMWLWSQTFCPVLYAWNDLGSRFWPRY VKVGSCFSKRSCSVPEGMVCKPSKSVHLTVLRWRCQRRGGQRC GWIPIQYPIISECKCSC | |  | | --- | |  | | quickGene  Sequence based search of  Entrez Gene data | | |  |  |  |  | | --- | --- | --- | --- | |  |  |  |  | | Homo sapiens | 100.0% |  | NOG | | Rattus norvegicus | 99.14% |  | Nog | | Mus musculus | 99.14% |  | Nog | | Gallus gallus | 82.59% |  | NOG | | Xenopus laevis | 78.13% |  | nog-A | |
|  |  | other quickApps    |  |  |  | | --- | --- | --- | |  | quickImage |  | |  | quickLit | *Expression of a Xenopus homolog of Brachyury (T) is an immediate-early response to mesoderm induction*  **Cell 67 (1), 79-87 (1991)** Smith, J.C., Price, B.M., Green, J.B., Weigel, D. and Herrmann, B.G. | |  |  |  | |
