# Supplementary material for: Evading the annotation bottleneck: using sequence similarity to search non-sequence gene data
Source: BMC Bioinformatics. 2008 Oct 17;9:442. doi: 10.1186/1471-2105-9-442 (PMC2587480; doi:10.1186/1471-2105-9-442)
Supplement: Additional file 1 — archive of code for the applications described in the manuscript. quick-release-archive. [file 1471-2105-9-442-S1.zip › quick-release-code/apache/htdocs/quickLit/index.html]

quickLit


|  |  |  |
| --- | --- | --- |
|  |  |  |
| >AAH67315.1  EMIVTKNGRRMFPVLKISVTGLDPNAMY SFLMDFVTADNNRWKYVNGEWVPGGKPE PQAPSCVYIHPDSPNFGAHWMKAPVSFS KVKLTNKMNGEGQIMLNSLHKYEPRIHI VRVGGPQKMITSHSFPETQFIAVTAYQN EEITALKIKHNPFAKAFLDAKERSDHKD FIDDAENGQQSGYSQLGNWLIPGTGSLC SSSNHHSQFGAPLSIPSSHGCERYTTLR NHRSSPYPSPYTHRNN | |  | | --- | |  | | quickLit  Sequence based literature search | | |  | | --- | | **Expression of a Xenopus homolog of Brachyury (T) is an immediate-early response to mesoderm induction** | | Cell 67 (1), 79-87 (1991) | | Smith, J.C., Price, B.M., Green, J.B., Weigel, D. and Herrmann, B.G. | ||  |  | other quickApps    |  |  |  | | --- | --- | --- | |  | quickImage |  | |  | quickGene | *Xenopus tropicalis* 100.0% **MGC76084**  *Xenopus laevis* 96.67% **bra3-a**  *Gallus gallus* 85.42% **T**  *Rattus norvegicus* 84.17% **T\_predicted** | |  |  |  | |
