# Supplementary figures and images for: Evading the annotation bottleneck: using sequence similarity to search non-sequence gene data
Source: BMC Bioinformatics. 2008 Oct 17;9:442. doi: 10.1186/1471-2105-9-442 (PMC2587480; doi:10.1186/1471-2105-9-442)

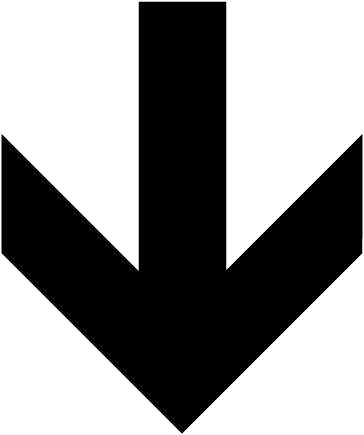

Supplement: Additional file 1 — archive of code for the applications described in the manuscript. quick-release-archive. [file 1471-2105-9-442-S1.zip › quick-release-code/apache/htdocs/resources/arrow-down-black-on-white.gif]

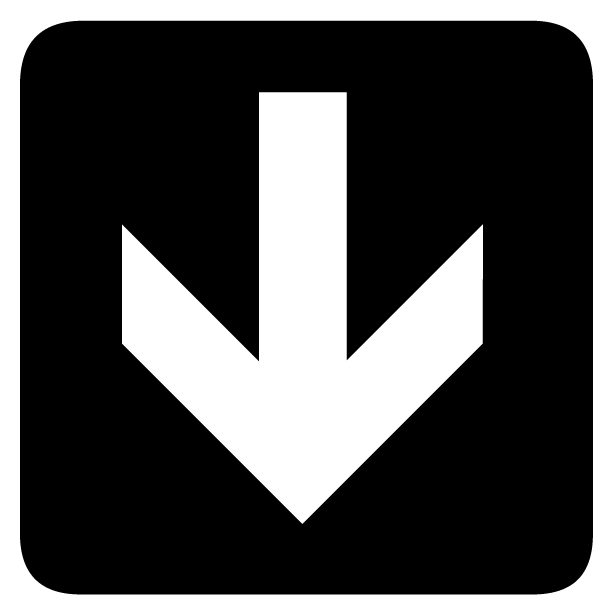

Supplement: Additional file 1 — archive of code for the applications described in the manuscript. quick-release-archive. [file 1471-2105-9-442-S1.zip › quick-release-code/apache/htdocs/resources/arrow-down-white-on-black.gif]

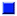

Supplement: Additional file 1 — archive of code for the applications described in the manuscript. quick-release-archive. [file 1471-2105-9-442-S1.zip › quick-release-code/apache/htdocs/resources/blue-but.gif]

Supplement: Additional file 1 — archive of code for the applications described in the manuscript. quick-release-archive. [file 1471-2105-9-442-S1.zip › quick-release-code/apache/htdocs/resources/blue.gif]

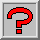

Supplement: Additional file 1 — archive of code for the applications described in the manuscript. quick-release-archive. [file 1471-2105-9-442-S1.zip › quick-release-code/apache/htdocs/resources/button-help.gif]

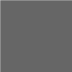

Supplement: Additional file 1 — archive of code for the applications described in the manuscript. quick-release-archive. [file 1471-2105-9-442-S1.zip › quick-release-code/apache/htdocs/resources/fl-0.gif]

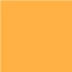

Supplement: Additional file 1 — archive of code for the applications described in the manuscript. quick-release-archive. [file 1471-2105-9-442-S1.zip › quick-release-code/apache/htdocs/resources/fl-1.gif]

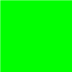

Supplement: Additional file 1 — archive of code for the applications described in the manuscript. quick-release-archive. [file 1471-2105-9-442-S1.zip › quick-release-code/apache/htdocs/resources/fl-2.gif]

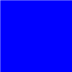

Supplement: Additional file 1 — archive of code for the applications described in the manuscript. quick-release-archive. [file 1471-2105-9-442-S1.zip › quick-release-code/apache/htdocs/resources/fl-3.gif]

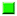

Supplement: Additional file 1 — archive of code for the applications described in the manuscript. quick-release-archive. [file 1471-2105-9-442-S1.zip › quick-release-code/apache/htdocs/resources/green-but.gif]

Supplement: Additional file 1 — archive of code for the applications described in the manuscript. quick-release-archive. [file 1471-2105-9-442-S1.zip › quick-release-code/apache/htdocs/resources/green.gif]

Supplement: Additional file 1 — archive of code for the applications described in the manuscript. quick-release-archive. [file 1471-2105-9-442-S1.zip › quick-release-code/apache/htdocs/resources/PI-100.gif]

Supplement: Additional file 1 — archive of code for the applications described in the manuscript. quick-release-archive. [file 1471-2105-9-442-S1.zip › quick-release-code/apache/htdocs/resources/PI-45.gif]

Supplement: Additional file 1 — archive of code for the applications described in the manuscript. quick-release-archive. [file 1471-2105-9-442-S1.zip › quick-release-code/apache/htdocs/resources/PI-66.gif]

Supplement: Additional file 1 — archive of code for the applications described in the manuscript. quick-release-archive. [file 1471-2105-9-442-S1.zip › quick-release-code/apache/htdocs/resources/PI-79.gif]

Supplement: Additional file 1 — archive of code for the applications described in the manuscript. quick-release-archive. [file 1471-2105-9-442-S1.zip › quick-release-code/apache/htdocs/resources/PI-87.gif]

Supplement: Additional file 1 — archive of code for the applications described in the manuscript. quick-release-archive. [file 1471-2105-9-442-S1.zip › quick-release-code/apache/htdocs/resources/PI-92.gif]

Supplement: Additional file 1 — archive of code for the applications described in the manuscript. quick-release-archive. [file 1471-2105-9-442-S1.zip › quick-release-code/apache/htdocs/resources/PI-95.gif]

Supplement: Additional file 1 — archive of code for the applications described in the manuscript. quick-release-archive. [file 1471-2105-9-442-S1.zip › quick-release-code/apache/htdocs/resources/PI-97.gif]

Supplement: Additional file 1 — archive of code for the applications described in the manuscript. quick-release-archive. [file 1471-2105-9-442-S1.zip › quick-release-code/apache/htdocs/resources/PI-98.gif]

Supplement: Additional file 1 — archive of code for the applications described in the manuscript. quick-release-archive. [file 1471-2105-9-442-S1.zip › quick-release-code/apache/htdocs/resources/PI-99.gif]

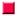

Supplement: Additional file 1 — archive of code for the applications described in the manuscript. quick-release-archive. [file 1471-2105-9-442-S1.zip › quick-release-code/apache/htdocs/resources/red-but.gif]

Supplement: Additional file 1 — archive of code for the applications described in the manuscript. quick-release-archive. [file 1471-2105-9-442-S1.zip › quick-release-code/apache/htdocs/resources/red.gif]

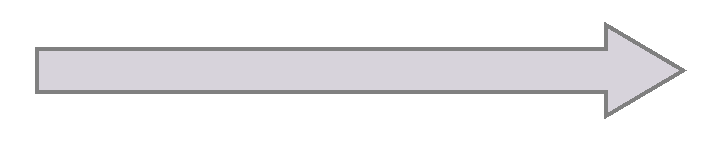

Supplement: Additional file 1 — archive of code for the applications described in the manuscript. quick-release-archive. [file 1471-2105-9-442-S1.zip › quick-release-code/apache/htdocs/resources/right-arrow-no-bg.gif]

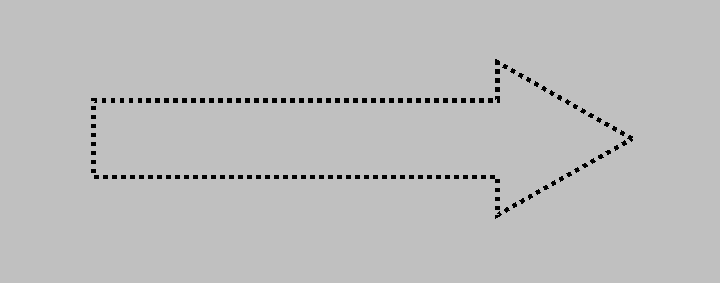

Supplement: Additional file 1 — archive of code for the applications described in the manuscript. quick-release-archive. [file 1471-2105-9-442-S1.zip › quick-release-code/apache/htdocs/resources/right-arrow.gif]

Supplement: Additional file 1 — archive of code for the applications described in the manuscript. quick-release-archive. [file 1471-2105-9-442-S1.zip › quick-release-code/apache/htdocs/resources/transparent.gif]

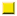

Supplement: Additional file 1 — archive of code for the applications described in the manuscript. quick-release-archive. [file 1471-2105-9-442-S1.zip › quick-release-code/apache/htdocs/resources/yellow-but.gif]

Supplement: Additional file 1 — archive of code for the applications described in the manuscript. quick-release-archive. [file 1471-2105-9-442-S1.zip › quick-release-code/apache/htdocs/resources/yellow.gif]
